# Paradoxical Air Embolism Without Patent Foramen Ovale During Craniotomy in the Sitting Position

**DOI:** 10.7759/cureus.4355

**Published:** 2019-04-01

**Authors:** Georgios A Maragkos, Justin Davanzo, S M Roberts, Brad E Zacharia

**Affiliations:** 1 Neurosurgery, Beth Israel Deaconess Medical Center, Boston, USA; 2 Neurosurgery, Penn State Milton S. Hershey Medical Center, Hershey, USA; 3 Anesthesiology, Penn State Milton S. Hershey Medical Center, Hershey, USA; 4 Neurosurgery, Penn State Health Milton S. Hershey Medical Center, Hershey, USA

**Keywords:** venous air embolism, patent foramen ovale, paradoxical air embolism

## Abstract

Craniotomy in the sitting position entails risk for venous air embolism (VAE). A 50-year-old male underwent pineal region mass resection through a sitting position craniotomy. Intraoperative transesophageal echocardiography confirmed the absence of intracardiac shunt. During craniotomy, VAE was noted inside the patient’s right heart, leading to hemodynamic instability. After repositioning to the supine position, significant crossover of air into the left heart was noted. Postoperatively, multiple small embolic strokes were noted. Patients who undergo craniotomy in the sitting position and are not found to have a patent foramen ovale (PFO) are not free of risk for paradoxical air embolism (PAE).

## Introduction

The sitting position is utilized when performing craniotomies on lesions in select locations but entails a risk of venous air embolism (VAE). In previous reports, this incidence has ranged anywhere from 9.3% to 76% [1-4]. In patients with a patent foramen ovale (PFO) or other intracardiac right-to-left shunts, there is an additional risk for paradoxical air embolism (PAE), which can result in end organ damage. We present a case of a patient undergoing a supracerebellar-infratentorial resection of a pineal tumor in the sitting position who developed a PAE, despite no evidence of PFO on pre-operative and intraoperative testing. This resulted in significant, albeit transient, neurologic deficits. Physiologic and technical considerations are discussed in hopes of providing a management paradigm to prevent and mitigate the effects of PAE.

## Case presentation

**Figure 1:**
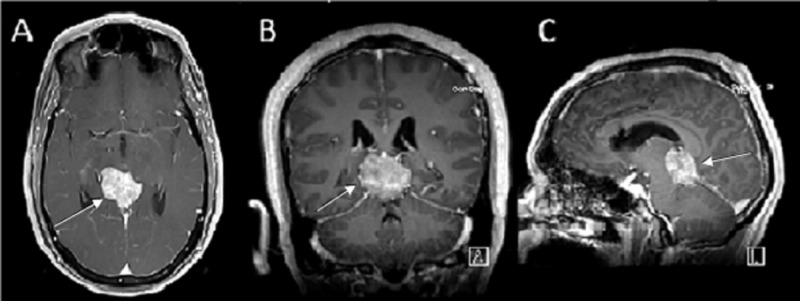
Gadolinium enhanced T1-weighted pre-operative magnetic resonance imaging (MRI) with (A) axial, (B) coronal and (C) sagittal view showing a homogenously enhancing lesion, the region of the pineal gland.

A 50-year-old Hispanic male was admitted with a three-day history of progressively worsening headaches. Computed tomography (CT) and magnetic resonance imaging (MRI) identified a pineal region mass measuring 3.5 x 2 x 3 cm (Figure 1).

A supracerebellar infratentorial approach in the sitting position was planned for resection of the pineal mass. Preoperatively, the patient was evaluated by transthoracic echocardiography (TTE) with agitated saline and Valsalva maneuver, to attempt to identify intracardiac shunts and none were identified.

**Figure 2:**
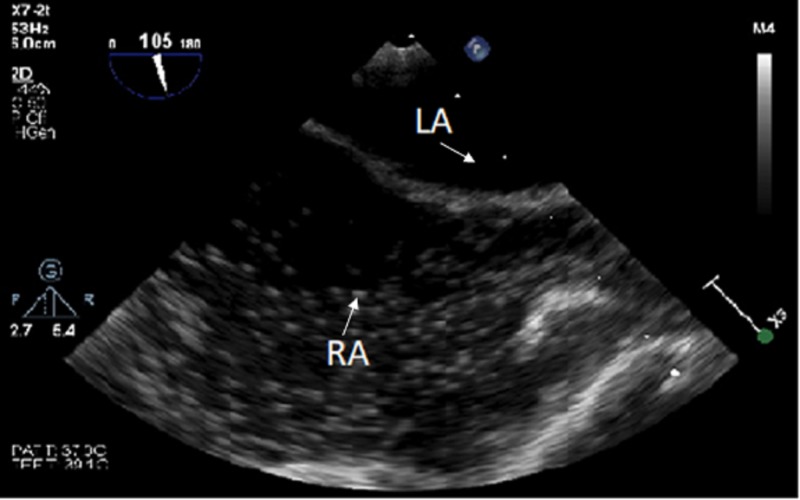
Pre-operative transesophageal echocardiogram, mid-esophageal bicaval view during agitated saline injection with Valsalva showing no evidence of intra-atrial shunt. RA: Right Atrium; LA: Left Atrium.

In the operating room, invasive arterial blood pressure monitoring, five-channel electrocardiogram (ECG), transesophageal echocardiogram (TEE), and a peripherally inserted central catheter (PICC) with the tip in the right atrium were placed. Following standard anesthetic induction and total intravenous maintenance consisting of propofol, dexmedetomidine, and remifentanil, an extensive TEE was performed in the supine position using contrast-enhanced ultrasound with agitated saline during a simulated Valsalva maneuver to rule out any possible right-to-left intracardiac shunts, including PFO, atrial septal defect (ASD) or ventricular septal defect (VSD) (Figure 2).

After confirming that no defect was present, the patient was placed in three-point pin fixation and was placed in the sitting position. Intracardiac shunt testing via TEE was repeated once again after the final position was reached. Again, no evidence of intracardiac shunt was noted.

**Figure 3:**
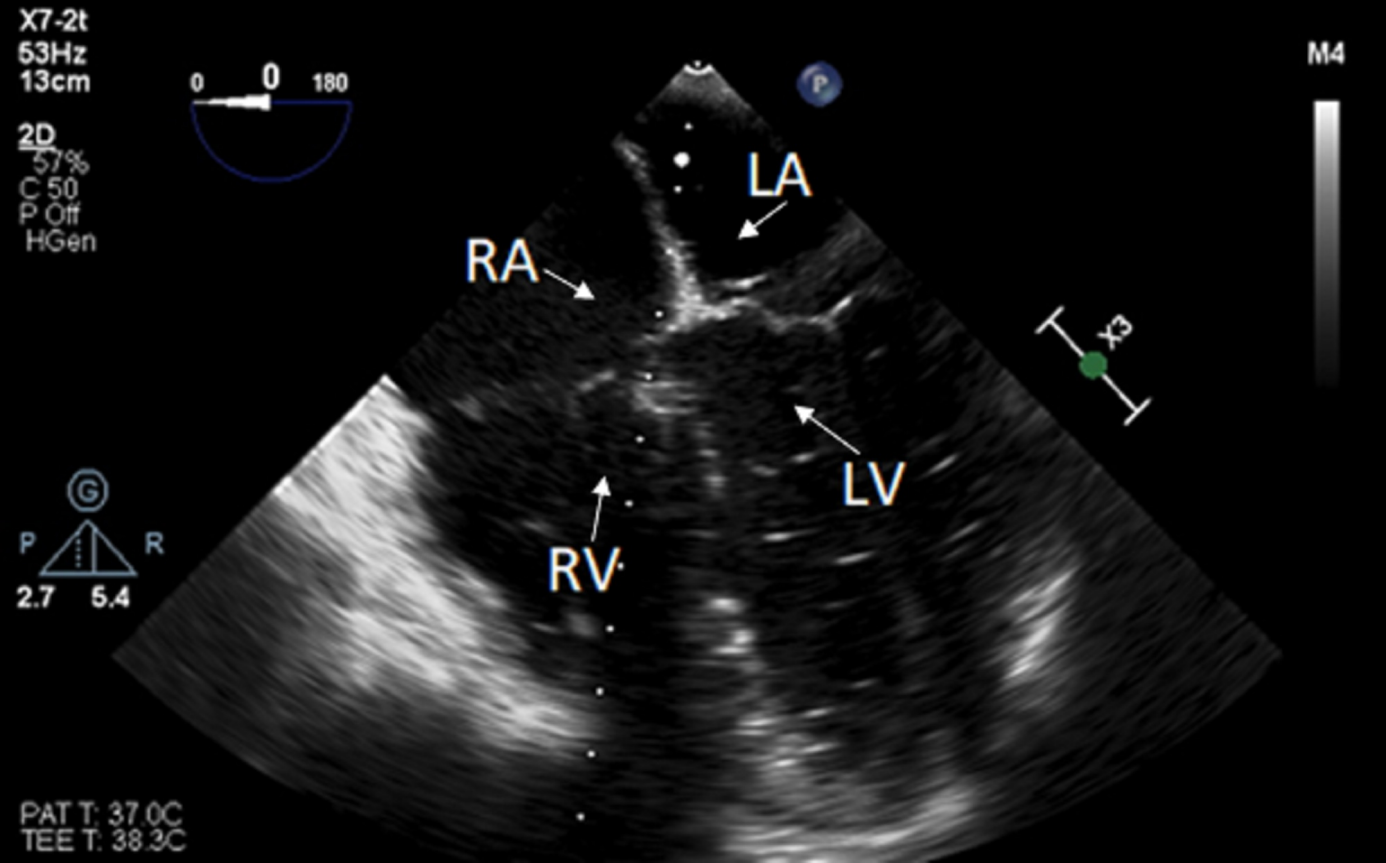
Intraoperative transesophageal echocardiogram, mid-esophageal 4-chamber view showing entrainment of air into the left-sided chambers despite a lack of intracardiac shunts identified. RA: Right Atrium; LA: Left Atrium; RV: Right Ventricle; LV: Left Ventricle.

Upon drilling of our initial burr hole, a small amount of air entrainment was noted on TEE. The operative field was flooded with irrigation which improved this; however, it did not completely resolve. The burr hole was waxed, and the patient remained hemodynamically stable. As further burr holes were drilled, air continued to entrain. We then completed our craniotomy, and elevated the bone flap. At this point, a large VAE was noted on TEE. The bone edges were quickly waxed, and continuous irrigation was performed. The patient was then noted to become acutely hypotensive with a coincident precipitous drop in end-tidal CO2 from 30 mm Hg to 21 mm Hg, despite unchanged pulse oximetry reading 100%. The field was covered with a wet lap pad and the head of the bed was lowered and rotated slightly to the left. Hemodynamics were supported with repeated doses of epinephrine 10-50 mcg. The fractional inspired oxygen (FiO2) was increased to one. Air was also aspirated via the previously placed PICC. Despite elevated right atrial pressures, no intracardiac shunt was noted. After hemodynamic recovery, air was noted on the left side of the heart (Figure 3).

This finding led to a presumptive diagnosis of right-to-left transpulmonary arteriovenous shunting allowing transmission of air during the transition to the supine position, solidified by increases in left atrial air during a Valsalva maneuver. The patient remained in the supine position until the left ventricle was free of air. A norepinephrine infusion was initiated to maintain a mean arterial pressure greater than 80 mm Hg. After the patient was stabilized, a discussion was made about continuing with the operation. Electroencephalography (EEG), motor-evoked potentials and somatosensory-evoked potentials remained stable. Considering the patient’s worsening pre-operative condition, the decision was made to proceed with the case. The patient was returned to the sitting position and the planned surgical procedure was completed resulting in gross total resection of the pineal mass (histologic diagnosis of pineal parenchymal tumor of intermediate differentiation).

**Figure 4:**
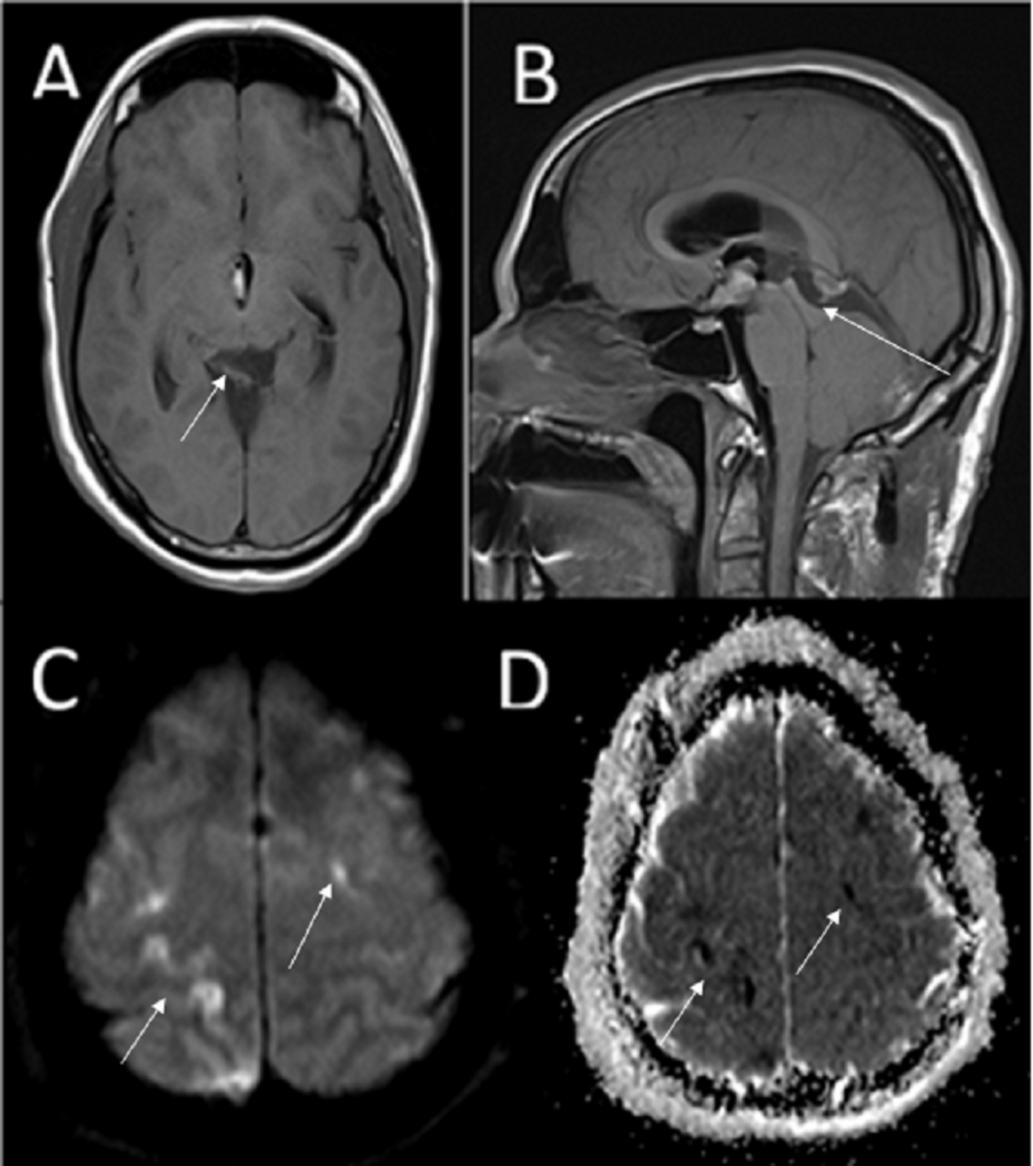
Post-operative magnetic resonance imaging (MRI). (A) Axial T1-weighted image and (B) sagittal T1-weighted image showing resection of the pineal lesion. (C) Diffusion-weighted imaging showing areas of restricted diffusion in the right and left frontal lobes and (D) the corresponding apparent diffusion coefficient (ADC) map confirming this finding. We were unable to obtain contrast-enhanced imaging secondary to patient condition.

Postoperatively, the patient remained intubated and underwent a CT scan of the head, which revealed air inside the ventricular system and the superior sagittal sinus. He was admitted to the neurological intensive care unit (ICU). On post-operative exam, upward gaze palsy, right gaze preference, left neglect and left hemiplegia were observed. CT angiography and perfusion showed no perfusion mismatch, but subsequent MRI of the brain showed acute cortical infarcts in the right frontal and parietal lobes involving the pre- and postcentral gyri (Figure 4).

There were also small acute infarcts in the left frontal lobe and the vermis. Systolic blood pressure was maintained between 100 and 160 mm Hg. The patient was evaluated for possible seizure activity with continuous EEG which was negative. The patient was successfully extubated and placed on a nonrebreather mask for 24 hours to speed resolution of his pneumocephalus. Over the following days, his left-sided weakness and neglect resolved completely. He was discharged to an inpatient rehabilitation facility with upward gaze palsy, but otherwise intact on POD six.

## Discussion

To the authors’ knowledge, this is the first report of a patient developing transient neurological deficits due to VAE in the absence of an intracardiac right-to-left shunt during a sitting position craniotomy. This highlights the potential of large-volume venous air emboli to cross over to the systemic circulation and cause PAE resulting in infarction. Patients with a PFO are at risk for PAE when placed in the sitting position, therefore routine preoperative evaluation with TTE and TEE is recommended [4]. According to the available literature, the bubble test that was utilized during the TEE has a sensitivity of 89.2% and a specificity of 91.4% [5].

PAE is extremely rare when no intracardiac shunt has been identified, but has been reported following non-neurosurgical cases [6-8]. In 2012, Schlundt et al. reported a case of left ventricular air crossover in the absence of a PFO in a neurosurgical patient undergoing resection of a cerebellopontine angle lesion in the sitting position [9]. In that case, the patient was immediately placed supine and the operation was completed in the lateral position. No neurological sequelae were observed. In our case, after the VAE cleared, we reverted to the sitting position and no further VAEs were identified. However, the patient experienced PAE with transient neurologic deficits.

Several possible explanations have been previously postulated, regarding passage of air emboli to the arterial circulation in the absence of a detectable intracardiac shunt. Transpulmonary passage of air is one of the proposed theories. Alternatively, hyperdynamic circulation in the presence of high alveolar-arterial partial oxygen pressure may facilitate opening of nascent intrapulmonary shunts [10]. Moreover, pulmonary capillaries are capable of filtering small amounts of air from the circulation to the alveoli. Substantial volumes of air, however, may eventually cause functional occlusion of the pulmonary capillaries and increased pulmonary arterial pressure, resulting in the passage of air into the systemic circulation [11-13]. Hypoxia has also been proposed as a mechanism to recruit intrapulmonary shunting. It has been shown that breathing 100% oxygen for one to two minutes can substantially decrease or eliminate intrapulmonary shunting for a period of time [14]. Therefore, it is postulated that a higher FiO2 during craniotomies in the sitting position may be utilized to help decrease the risk for PAE. Lastly, retrograde air ascension into the cerebral venous system is another possible mechanism of cerebral VAE in the absence of a PFO [15].

Interestingly, while our patient developed detectable air in the right atrium as soon as the craniotomy was performed, it was not until he was repositioned to the supine position that left ventricular air was noted. In the sitting position, there is higher perfusion of the lung bases compared to the apices. However, when placed in the supine position, perfusion of the apices is restored, and any potential underlying arteriovenous shunting becomes clinically relevant. Additionally, in the sitting position, air is trapped rostrally inside the right atrium; when moved to the supine position, the trapped air moves ventrally to the right ventricle, resulting in a higher air load finding passage through the pulmonary circulation.

## Conclusions

The case presented here illustrates the surgical and anesthesiologic considerations for craniotomy in the sitting position in the absence of a PFO on preoperative echocardiography. Such patients are not free of risk for PAE, which may occur in cases of large air load in the pulmonary circulation and during repositioning to the supine position. Here we emphasize the use of intraoperative TEE monitoring to immediately identify VAE, placement of central venous catheter in the right atrium to attempt aspiration of such air, and high FiO_2_ to avoid hypoxia and minimize potential intrapulmonary shunting. The possibility of pulmonary arteriovenous fistulas should also be considered. Identification of significant air load in the pulmonary circulation or frank PAE should prompt wound irrigation and transfer to the supine position to protect against further entrainment of air. While our patient did suffer from a temporary neurological deficit, no permanent deficits were noted.
